# Integrated analysis reveals critical cisplatin-resistance regulators E2F7 contributed to tumor progression and metastasis in lung adenocarcinoma

**DOI:** 10.1186/s12935-024-03366-6

**Published:** 2024-05-17

**Authors:** Xiaomin Mao, Shumin Xu, Huan Wang, Peng Xiao, Shumin Li, Jiaji Wu, Junhui Sun, Cheng Jin, Mo Shen, Yueli Shi, Bufu Tang, Ying Yang, Weiyu Chen, Zhiyong Xu, Yun Xu

**Affiliations:** 1 Department of Nursing, The Fourth Affiliated Hospital, Zhejiang University School of Medicine, Zhejiang University, Yiwu, 322000 China; 2 Department of Respiratory and Critical Care Medicine, Center for Oncology Medicine, the Fourth Affiliated Hospital of School of Medicine, and International School of Medicine, International Institutes of Medicine, Zhejiang University, Yiwu, 322000 China; 3 School of Medicine, Zhejiang University, Hangzhou, 310058 China; 4 Department of Respiratory and Critical Care Medicine, The First Affiliated Hospital of Wenzhou Medical University, Wenzhou, 325015 China; 5 Department of Reproductive Medicine Center, The First Affiliated Hospital of Wenzhou Medical University, Wenzhou, 325015 China; 6 Wuxi Center for Disease Control and Prevention, The Affiliated Wuxi Center for Disease Control and Prevention of Nanjing Medical University, Wuxi, 214023 China

**Keywords:** Lung adenocarcinoma, Cisplatin resistance, Prognostic, Immune infiltration, E2F7

## Abstract

**Background:**

Drug resistance poses a significant challenge in cancer treatment, particularly as a leading cause of therapy failure. Cisplatin, the primary drug for lung adenocarcinoma (LUAD) chemotherapy, shows effective treatment outcomes. However, the development of resistance against cisplatin is a major obstacle. Therefore, identifying genes resistant to cisplatin and adopting personalized treatment could significantly improve patient outcomes.

**Methods:**

By examining transcriptome data of cisplatin-resistant LUAD cells from the GEO database, 181 genes associated with cisplatin resistance were identified. Using univariate regression analysis, random forest and multivariate regression analyses, two prognostic genes, E2F7 and FAM83A, were identified. This study developed a prognostic model utilizing E2F7 and FAM83A as key indicators. The Cell Counting Kit 8 assay, Transwell assay, and flow cytometry were used to detect the effects of E2F7 on the proliferation, migration, invasiveness and apoptosis of A549/PC9 cells. Western blotting was used to determine the effect of E2F7 on AKT/mTOR signaling pathway.

**Results:**

This study has pinpointed two crucial genes associated with cisplatin resistance, E2F7 and FAM83A, and developed a comprehensive model to assist in the diagnosis, prognosis, and evaluation of relapse risk in LUAD. Analysis revealed that patients at higher risk, according to these genetic markers, had elevated levels of immune checkpoints (PD-L1 and PD-L2). The prognostic and diagnosis values of E2F7 and FAM83A were further confirmed in clinical data. Furthermore, inhibiting E2F7 in lung cancer cells markedly reduced their proliferation, migration, invasion, and increased apoptosis. In vivo experiments corroborated these findings, showing reduced tumor growth and lung metastasis upon E2F7 suppression in lung cancer models.

**Conclusion:**

Our study affirms the prognostic value of a model based on two DEGs, offering a reliable method for predicting the success of tumor immunotherapy in patients with LUAD. The diagnostic and predictive model based on these genes demonstrates excellent performance. In vitro, reducing E2F7 levels shows antitumor effects by blocking LUAD growth and progression. Further investigation into the molecular mechanisms has highlighted E2F7’s effect on the AKT/mTOR signaling pathway, underscoring its therapeutic potential. In the era of personalized medicine, this DEG-based model promises to guide clinical practice.

**Supplementary Information:**

The online version contains supplementary material available at 10.1186/s12935-024-03366-6.

## Introduction

Lung cancer is the leading cause of cancer-related deaths globally, with more than a million fatalities each year [1]. Non-small cell lung cancer (NSCLC), which includes about 85% of all lung cancer cases, is primarily lung adenocarcinoma (LUAD), accounting for nearly half of all lung cancer cases [2, 3]. Despite progress in chemotherapy, surgical techniques, and comprehensive treatments, the 5 year survival rate for advanced LUAD remains low, primarily due to postoperative metastasis and recurrence [4, 5].

Platinum-based chemotherapy, especially cisplatin, is a fundamental treatment for advanced NSCLC [6, 7]. Although used alongside other medications and radiotherapy, cisplatin’s effectiveness is significantly hampered by drug resistance [8]. Research indicates that cancer stem cells are resistant to cisplatin, contributing to tumor recurrence and metastasis [9]. Thus, investigating the mechanisms underlying cisplatin resistance and identifying molecular markers for early diagnosis, survival prediction, and relapse monitoring in LUAD are thus of paramount importance [10, 11]. In this light, bioinformatics serves as an essential tool for uncovering the molecular details of LUAD progression and pinpointing potential therapeutic targets.

Recent research emphasizes the critical role of cancer metabolism in not only supporting the growth and survival of tumors but also in influencing antitumor immune responses through metabolic byproducts and affecting the expression of immune molecules [12–14]. Specifically, the pathway involving cisplatin-induced activation of the glucocorticoid receptor, leading to resistance through microtubule-associated serine/threonine kinase 1 through the reprogramming of the mitogen-activated protein kinase (MAPK) pathway [15], has been highlighted. Cheng et al., utilizing high-throughput stimulated Raman scattering imaging and single-cell analysis, discovered that cisplatin-resistant cells exhibit increased fatty acid uptake along with reduced glucose uptake and lipogenesis. This indicates a shift from glucose-based to fatty acid-dependent metabolism for both anabolism and energy [16]. Furthermore, studies have shown that glutathione peroxidase-2 (GPX2) is significantly overexpressed in the cisplatin-resistant A549 cell line. Suppressing GPX2 reduces the activities of GPX and superoxide dismutase, as well as ATP production and glucose uptake in A549 drug-resistant cells, leading to an increase in malondialdehyde and reactive oxygen species. This results in inhibited cell proliferation and tumor growth [17]. Additionally, the relationship between thiamine and the p53/P21 axis, affecting the anti-proliferative effectiveness of cisplatin in LUAD cells through the modulation of 2-oxyglutarate/glutamate metabolism [18], has been explored. These insights suggest that exploring the link between cisplatin resistance and tumor metabolism in LUAD is a promising path toward developing more effective treatments.

The dynamic between the innate and adaptive immune systems significantly influences the tumor microenvironment through continuous immune surveillance [19]. This environment consists of a complex network of cells, including tumor cells, fibroblasts, immune cells, and endothelial cells [20–23]. Cancer cells often evade anti-tumor immune responses by upregulating inhibitory molecules such as cytotoxic T lymphocyte-associated antigen-4 (CTLA4) and programmed death-1 (PD-1)/programmed death-ligand 1 (PD-L1), as well as suppressive cytokines. These factors collectively lead to the inactivation of tumor-infiltrating T cells, thus preventing them from targeting adjacent tumor cells [24–28]. Currently, platinum-based chemotherapy combined with immune checkpoint inhibitors is the standard treatment for patients with LUAD. The use of anti-PD-1/PD-L1 monoclonal antibodies with platinum-based chemotherapy represents a significant advancement in treating metastatic NSCLC, designed to overcome resistance to single-agent therapy. Clinical trials have demonstrated that this combined treatment is more effective than chemotherapy alone in patients with mNSCLC, regardless of their cancer characteristics [6, 8], suggesting a wide applicability and potential for improved patient outcomes in this challenging field of oncology.

In studies on lung tumor models, the combined use of MEK inhibitors (MEKi) and cisplatin/pemetrexed has been shown to enhance the recruitment of CD8 T cells through CXCL10 secretion by cancer cells, thus improving the effectiveness of immune checkpoint inhibitors (ICIs). This combination not only promotes mitochondrial autophagy in an optineurin-dependent manner but also triggers CXCL10 production, which is stimulated by mitochondrial DNA and Toll-like receptor 9 (TLR9). Blocking TLR9 or autophagy/mitochondrial autophagy compromises the therapy's antitumor efficacy [8]. Moreover, Ginsenoside Rg3 shows promise in reducing the growth of cisplatin-resistant A549/DDP cells and their resistance to cisplatin by diminishing cisplatin-induced PD-L1 expression, which enhances T cell cytotoxicity against cancer cells [29]. These findings underscore the importance of developing an immunoassay model for patients with LUAD who have cisplatin resistance to assess the effectiveness of immune checkpoints comprehensively, leading to more precise and efficient treatment approaches.

This research analyzed 181 cisplatin resistance genes in LUAD using the TCGA and GEO databases. Cox regression analysis revealed that E2F transcription factor 7 (E2F7) and Family with sequence similarity 83 member A (FAM83A) was crucial for predicting LUAD diagnosis and prognosis. This study also delved into the connections between cisplatin resistance, tumor metabolism, and immune checkpoints in LUAD, developing a prognostic model that assesses the risk of negative outcomes. Additionally, this study explored the effect of E2F7 knockdown affects tumor growth and metastasis, both in vivo and in vitro, aiming to improve the early detection of cisplatin-resistant LUAD and support tailored treatment strategies.

## Materials and methods

### Screening of cisplatin-resistant related differentially expressed genes

The raw data of gene expression were sourced from GSE21656, a dataset on cisplatin resistance in NCI-H460 cells. In brief, H460 cells were treated with 3 µM cisplatin for seven days, with the surviving cells categorized as drug-resistant. Differentially expressed genes (DEGs) were identified between cisplatin-resistant and non-resistant cells using the “limma” R package (version 3.5.1), applying a log2-fold change (FC) ≥ 1 and an adjusted P-value < 0.05 as the selection criteria.

### Pathway enrichment analysis of the DEGs

To investigate the potential functions of these DEGs, GO and KEGG pathway enrichment analyses were conducted utilizing public databases: OmicsBean (www.omicsbean.cn/), the Database for Annotation, Visualization, and Integrated Discovery (DAVID, https://david.ncifcrf.gov), and the Metascape database (http://metascape.org/) [30]. A P-value < 0.05 was the criterion for significantly enriched pathways, with a focus on the top 20 most significant signaling pathways.

### Prognostic related gene signature construction and survival prediction reliability evaluation

**Fig. 1:**
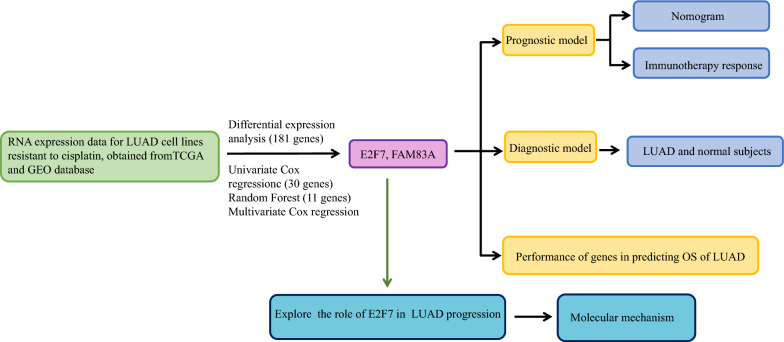
Process flowchart for analysis

The random forest algorithm was utilized to identify genes correlated with overall survival (OS) in patients. This process involved the use of the "random forest" in R package, setting the number of random forest decision trees to 500, and applying the default parameters. Subsequently, a prognostic risk score for the DEGs identified by the Random Forest algorithm was developed using multivariate Cox regression analysis. The formula for the risk score is as follows: risk score = [Expression level of Gene 1 × coefficient] + [Expression level of Gene 2 × coefficient] + … + [Expression level of Gene n × coefficient]. Patients with LUAD who had survival data were divided into high- and low-risk groups based on the median risk score. Kaplan–Meier (K–M) survival plots were created to compare the overall survival rates of the low- and high-risk groups, utilizing the “survival” package in R. The time-dependent receiver operating characteristic (ROC) curve analysis was conducted to assess the predictive capability of the gene signature, using the “survival ROC” package. Additionally, the GSE31210 and GSE30219 LUAD patient datasets, which include survival information, were retrieved from the GEO database for validation purposes (Fig. 1).

### Nomogram construction and validation

A predictive nomogram was developed to estimate the prognosis of patients with LUAD, integrating independent prognostic factors from multivariate Cox regression analyses, using the “rms” package in R. Calibration curves were generated to verify the nomogram's accuracy, with the proximity of these curves to the 45° reference line indicating the nomogram's prognostic prediction efficacy (nfold = 10). Time-dependent ROC curves were also plotted to evaluate the predictive accuracy and sensitivity of the nomogram compared with individual prognostic factors. The consistency index (C index) was calculated to measure the nomogram's predictive performance regarding OS.

### Gene set enrichment analysis

The DEGs related to metabolism were determined in the high- and low-risk groups. Gene set enrichment analysis (GSEA) was carried out, setting the significance threshold for enriched pathways at P < 0.05.

### Estimation of immune cells infiltrating

The correlation between immune cell infiltration and risk score was analyzed. The “CIBERSORT algorithm” package in R was used to assess the abundance of 22 types of immune cells in the low- and high-risk groups of patients with LUAD. The TCGA cohort served as the training set, with the GSE31210 and GSE30219 cohorts as validation sets. To explore the relationship between gene signature and immune checkpoint expression levels (PD-L1/PD-L2/IDO1/B7H3), crucial for tumor immune evasion, this study utilized sequences from the TCGA database, which were normalized before comparing the expression levels of these genes in high- and low-risk LUAD patient groups.

### Estimate the independent prognostic value of the two-gene signature

Univariate and multivariate Cox analyses identified whether the signature of cisplatin-resistant genes was a significant independent prognostic factor in predicting the OS of patients with LUAD, compared with other clinical parameters. A P-value of less than 0.05 was deemed statistically significant, with 95% confidence intervals (CIs) and hazard ratios (HRs) calculated for each variable.

### Diagnostic model establishment

A stepwise logistic regression analysis was conducted to establish a diagnostic model utilizing two genes associated with cisplatin resistance. The TCGA cohort served as the training set, and the GSE102287 cohort functioned as the validation set.

### Cell culture and transfection

Human NSCLC cell lines A549, PC9, and the mouse Lewis lung cancer cell line (LLC) were acquired from ATCC (Manassas, VA, USA). A549 and PC9 were cultured in Roswell Park Memorial Institute (RPMI) 1640 medium, whereas LLC was cultured in Dulbecco's modified Eagle medium (DMEM), both supplemented with 10% fetal bovine serum (FBS) and 1% penicillin–streptomycin-gentamicin (PSG) solution, in a 5% CO_2_ atmosphere at 37 °C. For gene silencing, two siRNA sequences targeting E2F7, “GCAAAUGGCCUACCUCCAATT” and “UUGGAGGUAGGCCAUUUGCTT”, were obtained from GenePharma (Shanghai, China). The siRNAs were delivered into cells using jetPRIME^®^ transfection reagent (Polyplus-transfection, Strasbourg, France). The transfection mixture was prepared by diluting 1 nmol of siRNA in 200 μL of jetPRIME^®^ buffer, followed by vortexing and brief centrifugation. Then, 4 μL of jetPRIME^®^ reagent was added to the mixture, vortexed, and incubated for 10 min at room temperature. Approximately 200 μL of this transfection mixture was added to each well of a 6-well plate when the cells reached 50% confluency. After 24 h of incubation, the cells were ready for subsequent experiments.

### Cisplatin-resistant A549 IC50 assay

Cisplatin-resistant A549 cells were transfected with either a non-coding control (NC) or E2F7-specific siRNA. Both NC and E2F7 knockdown cells were then plated in 96-well plates (2 × 10^3^ cells/100 μL per well) and treated with varying concentrations of cisplatin (10, 20, 40, and 80 μg/mL) for 24 h. The absorbance was measured at 450 nm using a Cytation microplate reader (Bio Tek, California, USA).

### Quantitative real-time PCR

Total RNA extraction was performed using TRIzol reagent (Thermo Fisher Scientific, Massachusetts, USA), followed by reverse transcription using PrimeScript^™^ RT Master Mix (Takara, Tokyo, Japan). Quantitative RT-PCR (qRT-PCR) was carried out with TB Green Premix Ex Taq^™^ (Takara, Tokyo, Japan) and specific primers (Supplementary Table 4).

### Cell viability assay and colony formation assays

Cell proliferation was assessed using the Cell Counting Kit-8 (CCK-8) assay. NC and E2F7 knockdown cells were plated in 96-well plates (2 × 10^3^ cells/100 μL per well) and incubated for 24, 48, 72, and 96 h. Then, 10 μL of CCK-8 solution (Beyotime, Beijing, China) was added to each well and incubated for an additional 2 h at 37 °C. Absorbance at 450 nm was measured using a Cytation microplate reader. For colony formation assays, cells were seeded in a 6-well plate at a density of 2 × 10^3^ cells and allowed to grow for two weeks. Colonies were then fixed with 4% paraformaldehyde and stained with 0.5% crystal violet. The number of colonies was determined using ImageJ software.

### 5-Ethynyl-2’-deoxyuridine (EdU) assay

Transfected and non-transfected cells were cultured in 12-well plates and incubated overnight. They were treated with 10 μM EdU (Beyotime, Beijing, China) for 2 h, then fixed with 4% paraformaldehyde at room temperature for 15 min. Following this, the cells were washed with phosphate-buffered saline (PBS) containing 5% bovine serum albumin and permeabilized with 0.5% Triton X-100 in PBS for 10 min. EdU detection was achieved using Alexa Fluor 488 through a click chemistry reaction, incubated in the dark for 30 min. The cells were then stained with Hoechst 33342 for 10 min and examined under a fluorescence microscope (BX53; Olympus, Tokyo, Japan).

### Cell apoptosis assay

After transfection, cells were seeded in 12-well plates and incubated for 24 h. The cells were collected, washed with PBS, and resuspended in binding buffer. Annexin V-FITC and propidium iodide (PI) were added for 10–15 min as per the Annexin V-FITC apoptosis detection kit's instructions (Beyotime, Beijing, China). Analysis was conducted using a flow cytometer Cytoflex S (Beckman, California, USA).

### Wound-healing and transwell assays

The monolayer of cells was scratched to a straight line in a 6-well plate, and incubated without serum for 24 h. The migration was recorded at 0 h and 24 h by BX53 (Olympus, Tokyo, Japan). Transwell assays were employed to detect cell migration and invasion ability. The cells were collected and suspended in a serum-free medium. About 600 μL of medium containing 10% FBS was added to a 24-well plate, and 200 μL cell suspension was added to the upper chamber, both with and without matrigel. After being incubated at 37 ℃ for 24 h, the chamber was fixed in 4% paraformaldehyde and stained with crystal violet. The images were observed under a microscope, and the number of migration and invasive cells were calculated by Image J.

### Western blotting

The total proteins were extracted from LUAD cell lines, and protein concentrations were detected by a BCA protein assay kit (Beyotime, Shanghai, China). Sodium dodecyl sulfate‐polyacrylamide electrophoresis (SDS‐PAGE) gel was employed to fractionate protein samples, which were subsequently transferred to a polyvinylidene fluoride (PVDF) membrane (Millipore, Massachusetts, USA). After blocking with 5% skim milk for 1 h, the membranes were incubated with primary antibodies overnight at 4 ℃ (Supplementary Table 5). Next, they were incubated with goat anti-rabbit or anti-mouse IgG, linked with HRP, for 1 h. The membranes were developed by chemiluminescence on a Chemidoc touch (BioRad, California, USA).

### Immunohistochemistry staining

Tissue samples were dewaxed and underwent antigen retrieval in sodium citrate for 20 min. After permeabilization and blocking, they were incubated with primary antibody overnight at 4 °C. Lung tissue sections were specifically stained with anti-E2F7 at 4 °C overnight, followed by secondary antibody and DAB staining. Paraffin sections from 10 LUAD tissue cases were obtained from The Second Affiliated Hospital of Zhejiang University School of Medicine. The study received approval from the institutional review committee of The Second Affiliated Hospital of Zhejiang University School of Medicine (approval number: 2019–466/I2019001631). All the patients have written informed consent before surgery.

### Animal experiments

Six-week-old C57 mice, sourced from SLAC (Shanghai, China), were used to establish a xenograft mouse model through subcutaneous injection of LLC cells (1 × 10^6^) transfected with shE2F7 or shNC. Tumor size was monitored every three days starting on day 6 post-injection. On day 18, the mice were euthanized, and tumor weights were recorded. For the pulmonary metastasis model, LLC cells (1 × 10^6^) were injected into the tail vein. After 20 days, mice were euthanized, and lung tissues were processed for H&E staining. All procedures were approved by the Zhejiang University School of Medicine's committee.

### Statistical analysis

Statistical analyses were conducted using Prism 7.0 (GraphPad, San Diego, CA, USA), comparing two groups with a two-sided Student’s two-sided t-test. The results were presented as mean ± SD, with p-values being two-tailed. A p-value < 0.05 was deemed statistically significant.

## Results

### DEG screening for cisplatin resistance in lung adenocarcinoma

**Fig. 2:**
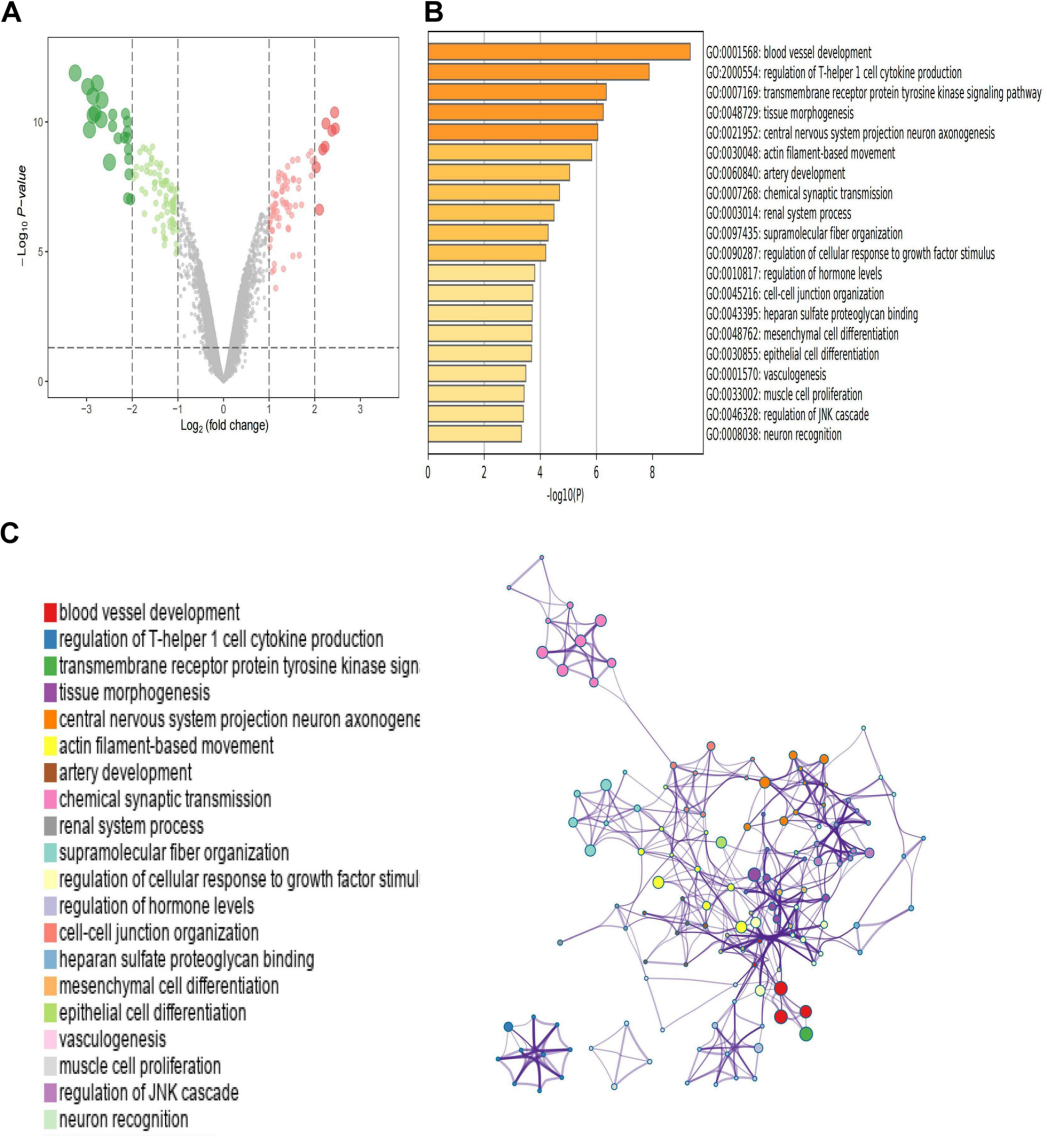
Identification and functional annotation of DEGs. **A** Volcano plot of upregulated and downregulated DEGs. **B**, **C**. GO and KEGG analyses of the genes in the differential network shown as a bar chart (**B**) and a functional annotation graph of protein-protein interactions (**C**)

In our study on cisplatin resistance in lung adenocarcinoma, the GSE21656 dataset was analyzed to compare cisplatin-resistant cells with their non-resistant counterparts. The findings, illustrated in a volcano plot (Fig. 2A), revealed 181 DEGs by employing a threshold of FDR < 0.05 and |log2 FC|≥ 1. In the cisplatin-resistant cell group, 73 genes were upregulated, marked in red, and 108 genes were downregulated, indicated in green. Detailed information on these DEGs for both cisplatin-resistant and non-resistant LUAD cohorts is provided in Supplementary Table 1.

Further examination through GO and KEGG analyses identified the top 20 significant pathways, depicted in a bar plot (Fig. 2B) and a network plot (Fig. 2C). These pathways include blood vessel development, regulation of T-helper 1 cell cytokine production, transmembrane receptor protein tyrosine kinase signaling pathway, tissue morphogenesis, and central nervous system projection neuron axonogenesis, all significantly affected.

Upon screening the DEGs, univariate regression analysis was applied to identify 30 genes associated with poor prognosis (HR > 1, P < 0.05; Supplementary Table 2). Subsequent analysis using a random forest curve pinpointed 11 genes with a significant effect on adverse prognosis, using a cutoff value of 0.2 (Supplementary Table 3). Among these, multivariate regression analysis (P < 0.05) singled out two genes, E2F7 and FAM83A, as critical to our predictive model, detailed in Table 1. This study established a risk score model as follows: Risk score = (0.256255 × expression level of E2F7) + (0.009532 × expression level of FAM83A). By using the median score value, patients with LUAD were categorized into the respective risk groups.

**Table 1:** Two cisplatin resistant-related genes were significantly associated with the OS of the LUAD patients

Gene Name	HR	HR.95L	HR.95H	P value
E2F7	1.292082965	1.164664198	1.433441838	1.31E-06
FAM83A	1.009578229	1.006864996	1.012298773	3.85E-12

### Survival prediction of patients with LUAD according to the gene signature

**Fig. 3:**
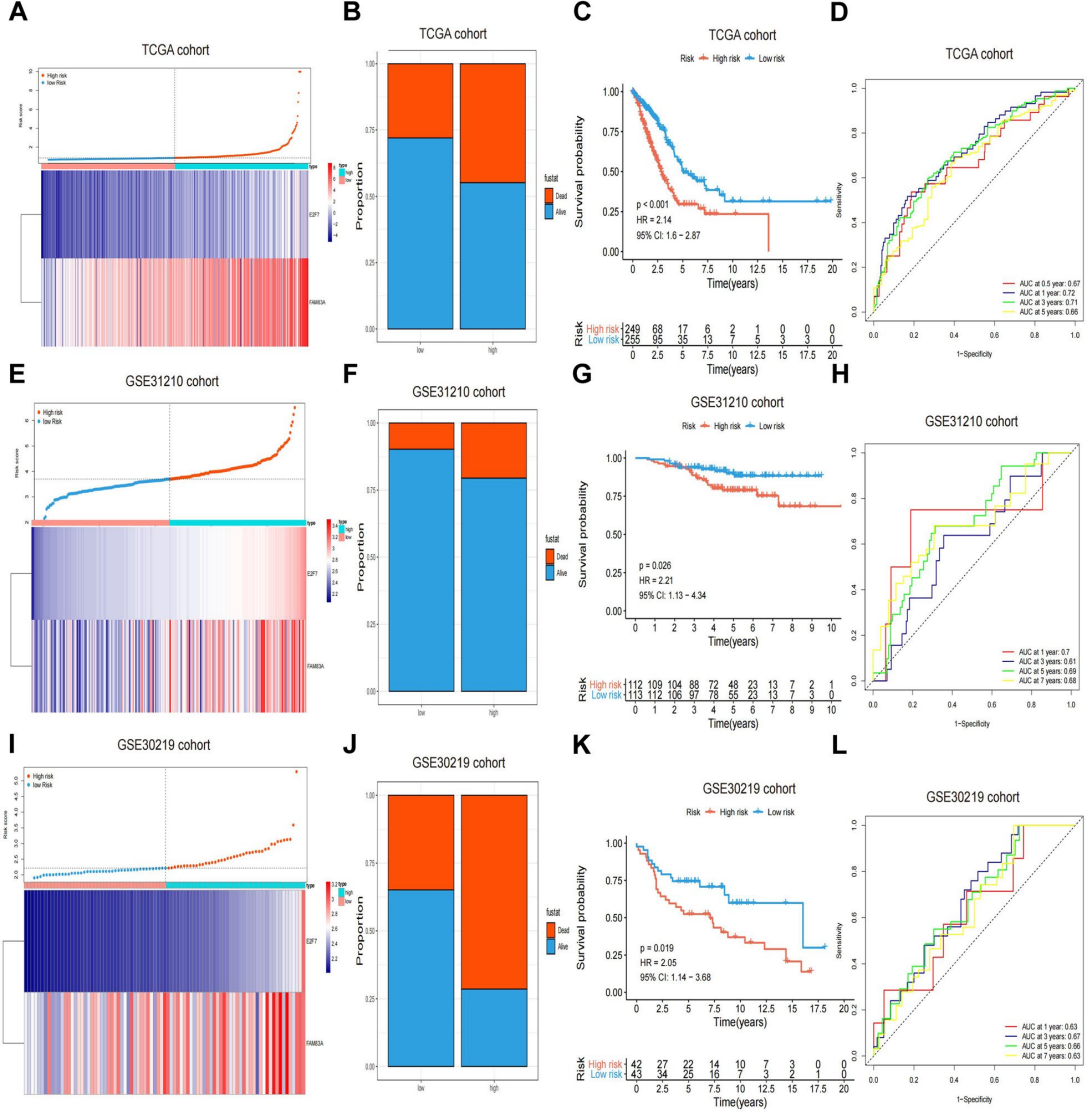
Kaplan-Meier survival analysis, risk score distribution, and time-dependent ROC curves of a prognostic model in the LUAD cohort from TCGA. **A**, **B** Distribution of risk score, survival status, and expression of two DEGs for patients in the low- and high-risk groups in the TCGA training set. **C**, **D** Kaplan-Meier survival analyses (**C**) and time-dependent ROC curve analyses (**D**) in the TCGA training set according to the risk score. **E**, **F** Distribution of risk score, survival status, and expression of two DEGs for patients in the low- and high-risk groups in the GSE31210 validation cohort. **G**, **H** Kaplan-Meier survival analyses and (**G**) time-dependent ROC curve analyses (**H**) in the GSE31210 validation cohort according to the risk score. **I**, **J** Distribution of risk score, survival status, and expression of two DEGs for patients in the low- and high-risk groups in the GSE30219 validation cohort. **K**, **L** Kaplan–Meier survival analyses and (**K**) time-dependent ROC curve analyses (**L**) in the GSE30219 validation cohort according to risk score

The median value was utilized as the cutoff to classify patients with LUAD into different risk groups. The risk score model was calculated as follows: Risk score = (0.256255 * expression level of E2F7) + (0.009532 * expression level of FAM83A). A heatmap demonstrated the distribution of clinical features, using the median value to segregate patients into high-risk and low-risk categories, as illustrated in Fig. 3A. Figure 3B shows a higher number of deceased patients in the high-risk group. Kaplan–Meier curve analysis revealed a significant association between risk scores and OS time, with low-risk patients having a considerably longer OS than those at higher risk (p < 0.0001; HR = 2.14; 95%CI 1.6–2.87) (Fig. 3C). The area under the curve (AUC) values for the 0.5, 1, 2, and 3 year ROC curves were 0.67, 0.72, 0.71, and 0.66, respectively, indicating the model's effectiveness in predicting survival time within this training dataset (Fig. 3D). These findings were corroborated using two related GEO datasets (i.e., GSE31210 cohort and GSE30219 cohort), with patients divided into high- and low-risk categories based on the median value. Importantly, the risk score among the deceased was higher than that among the living. The Kaplan–Meier curve suggested that patients with high-risk scores were linked to shorter survival times compared to individuals with low-risk scores (GSE31210 cohort [p = 0.026; HR = 2.2; 195% CI 1.13–4.34], GSE30219 cohort [p = 0.019; HR = 2.05; 195% CI 1.14–3.68]). The AUC areas at 1, 3, 5, and 7 years were 0.7, 0.61, 0.69, and 0.68 in the GSE31210 cohort and 0.63, 0.67, 0.66, and 0.63 in the GSE30219 cohort, respectively. This finding aligns with the results observed in the TCGA cohort findings (Fig. 3E, L). These results emphasize the predictive power of the risk score model, including E2F7 and FAM83A, for survival outcomes in patients with LUAD and highlight the prognostic significance of combined DEGs in determining LUAD survival prospects.

### Correlations between risk scores and tumor metabolic pathways

**Fig. 4:**
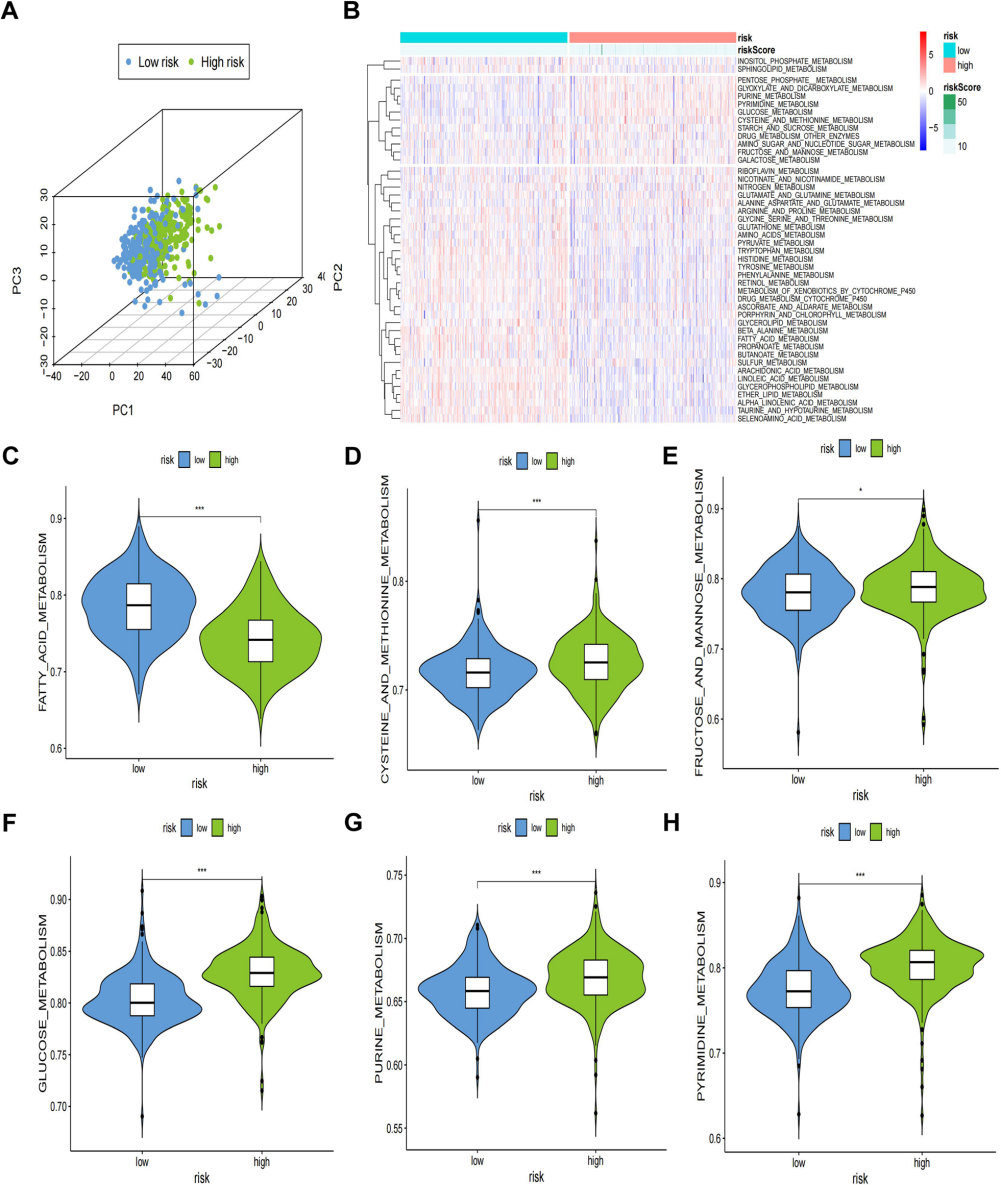
High- and low-risk groups exhibited distinct distribution patterns and varied gene-set enrichment results. **A** PCA results of the low- and high-risk groups based on the two gene signature. **B** Heatmap of the metabolic pattern between the low- and high-risk groups. **C** Comparison of fatty acid metabolism levels. **D** Comparison of cysteine and methionine metabolism levels. **E** Comparison of fructose and mannose metabolism levels. **F** Comparison of glucose metabolism levels. **G** Comparison of purine metabolism levels. **H** Comparison of pyrimidine metabolism levels

Subsequently, this study explored the association between varying risk scores and tumor metabolic pathways. Principal component analysis (PCA) was used to discern patterns between high- and low-risk patients, revealing distinct distributions as shown in Fig. 4A. Gene set enrichment analysis (GSEA), presented in Fig. 4B, confirmed the significant association between risk scores and tumor metabolism pathways. Violin plots (Fig. 4C–H) further clarified this association, showing enhanced gene enrichment in several tumor metabolic pathways, such as fatty acid, cysteine and methionine, fructose and mannose, glucose, and both purine and pyrimidine metabolism among high-risk patients. These findings underline the relevance of metabolic pathways in understanding tumor progression risk profiles.

### Analysis of immune cell infiltration and expression of immune checkpoints in patients with LUAD with different risk scores

**Fig. 5:**
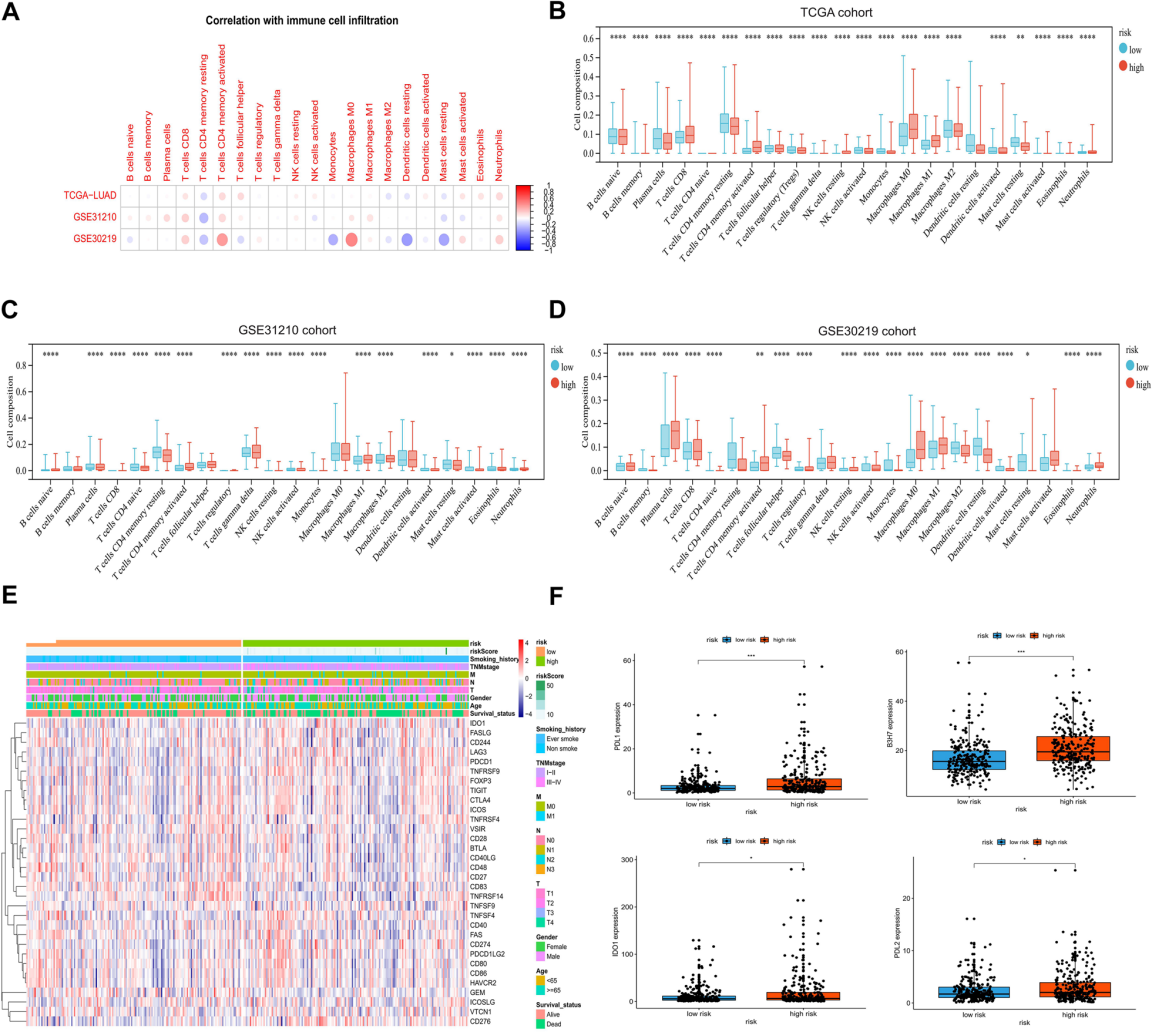
Correlations between low- and high-risk scores with immune infiltration and immune checkpoint. **A** Correlation between risk score and immune cell infiltration was determined by analyzing TCGA, GSE31210, and GSE30219 datasets. **B** Differences in the abundance of immune cell infiltration were defined by the two gene signatures in the TCGA training set. **C** Differences in the abundance of immune cell infiltration were defined by the two gene signatures in the GSE31210 validation cohort. **D** Differences in the abundance of immune cell infiltration defined by the two gene signatures in the GSE30219 validation cohort. **E** Heatmap of the expression levels of immune-related genes in the low- and high-risk groups. **F** Comparison of PD-L1, PD-L2, IDO1, and B7H3 expression levels

In examining the link between our prediction model and immune infiltration status, this study analyzed risk scores and 21 types of infiltrating immune cells across three cohorts: TCGA-LUAD, GSE32110, and GSE30219. The correlations were visually represented, with red points for positive and blue points for negative correlations. Despite variability in immune infiltration among the cohorts, consistent immune responses, including infiltration by CD4^+^ memory resting T cells and CD4 memory-activated T cells, were identified, as shown in Fig. 5A. Cohort-specific analyses revealed that patients with high risk scores in the TCGA cohort had increased infiltration of CD8^+^ T cells and CD4 memory-activated T cells (Fig. 5B), a trend consistent with the GSE31210 and GSE30219 cohorts (Fig. 5C, D). These findings suggest a connection between higher risk scores and increased immune activation in patients with LUAD.

Subsequently, this study investigated the relationship between the risk score and the expression levels of common immune checkpoints. The heatmap shown in Fig. 5E displays the distribution of these checkpoints among patients with varying risk levels. Notably, those in the high-risk group exhibited increased expression levels of significant immune checkpoints, including PD-L1, B7H3, IDO1, and PD-L2, as shown in Fig. 5F. This pattern indicates that patients with LUAD having higher risk scores may be more amenable to immunotherapy, underscoring the importance of personalized treatment strategies based on risk evaluation.

### Construction of the nomogram predicting overall survival for patients with LUAD in the TCGA cohort

**Fig. 6:**
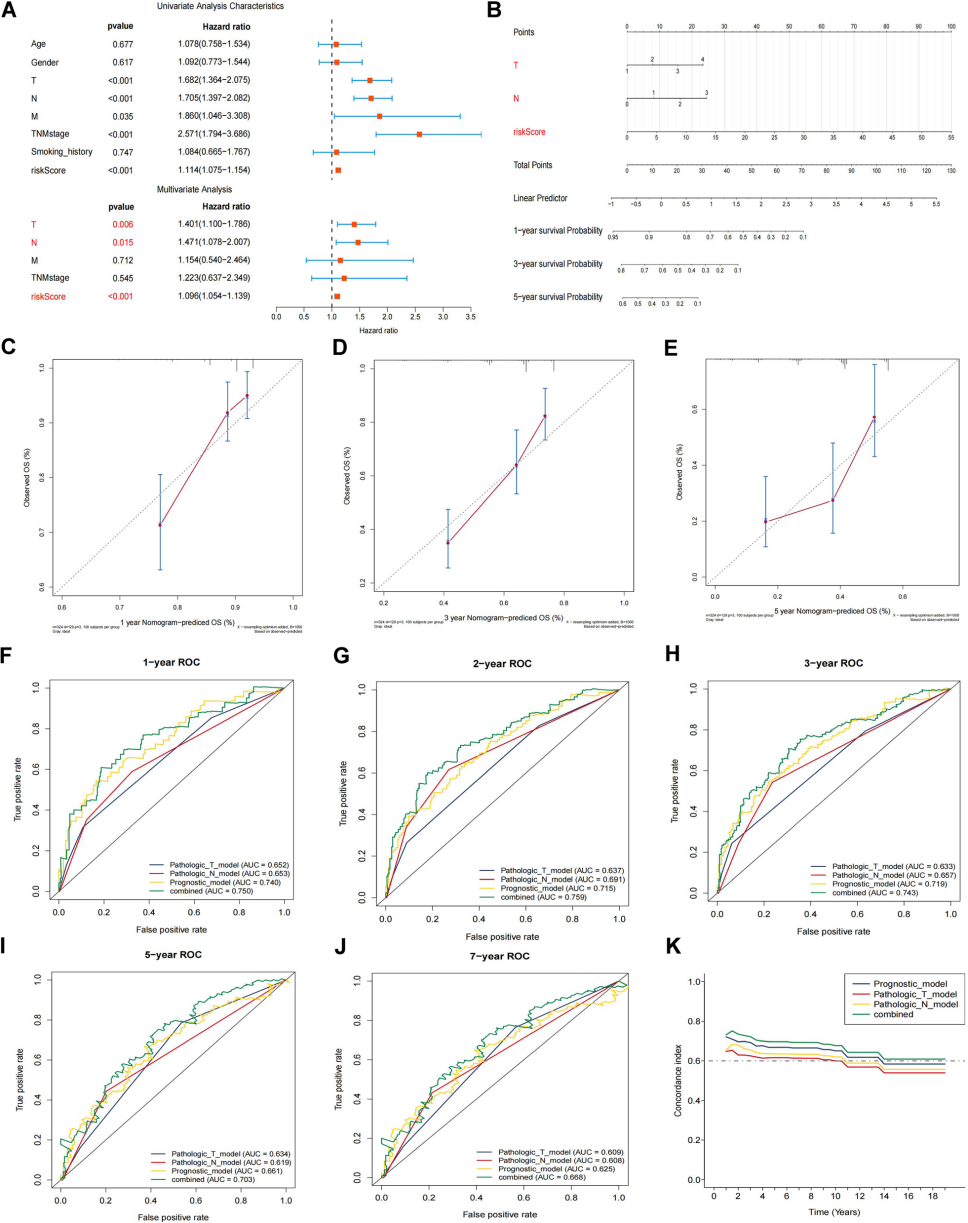
Development of a nomogram for predicting overall survival in patients with LUAD based on the TCGA cohort. **A** Univariate and multivariate Cox regression analyses shown as a forest plot of correlations between the two-gene signature and clinical characteristics with overall survival. **B** Nomogram plot for predicting 1-, 3-, and 5 year overall survival of patients with LUAD based on clinical information and risk score. **C**–**E** Calibration plots were built to assess the predictive accuracy of the nomogram at 1-, 3-, and 5 year overall survival. **F**–**J** Time-dependent ROC curves were drawn to compare the prognostic accuracy of T stage, N stage, risk score, and a combination of all three (1, 2, 3, 5, and 7 years). **K** Time-dependent C index built according to T stage, N stage, risk score, and a combination of all three

To verify the two-gene signature-derived risk score as an independent biomarker, COX proportional hazards regression analysis was applied on the TCGA cohort. The results, depicted in Fig. 6A, revealed that T stage (P = 0.006, HR = 1.401, 95% CI = 1.100–1.786), N stage (P = 0.015, HR = 1.471, 95% CI = 1.078–2.007), and the risk score (P < 0.001, HR = 1.096, 95% CI = 1.054–1.139) were significantly linked to the OS of patients with LUAD. This confirms the risk score as a unique prognostic factor for LUAD. To improve the precision of patient prognosis, a prognostic nomogram model incorporating significant factors from the multivariate analysis was developed. The results showed that the risk score was a more reliable survival predictor than T and N stages, as illustrated in Fig. 6B. The calibration curve demonstrated that the prediction probability of the nomogram closely matched actual outcomes (Figs. 6C–E). The AUCs for 1-, 2-, 3-, 5-, and 7 year predictions in the nomogram were 0.750, 0.759, 0.743, 0.703, and 0.668, respectively, proving the model’s accuracy and reliability (Figs. 6F–J). Moreover, the model’s concordance index (C-index) exceeded those of other individual indices, highlighting its superior predictive power (Fig. 6K).

### Construct a diagnostic model for distinguishing LUAD from normal samples

**Fig. 7:**
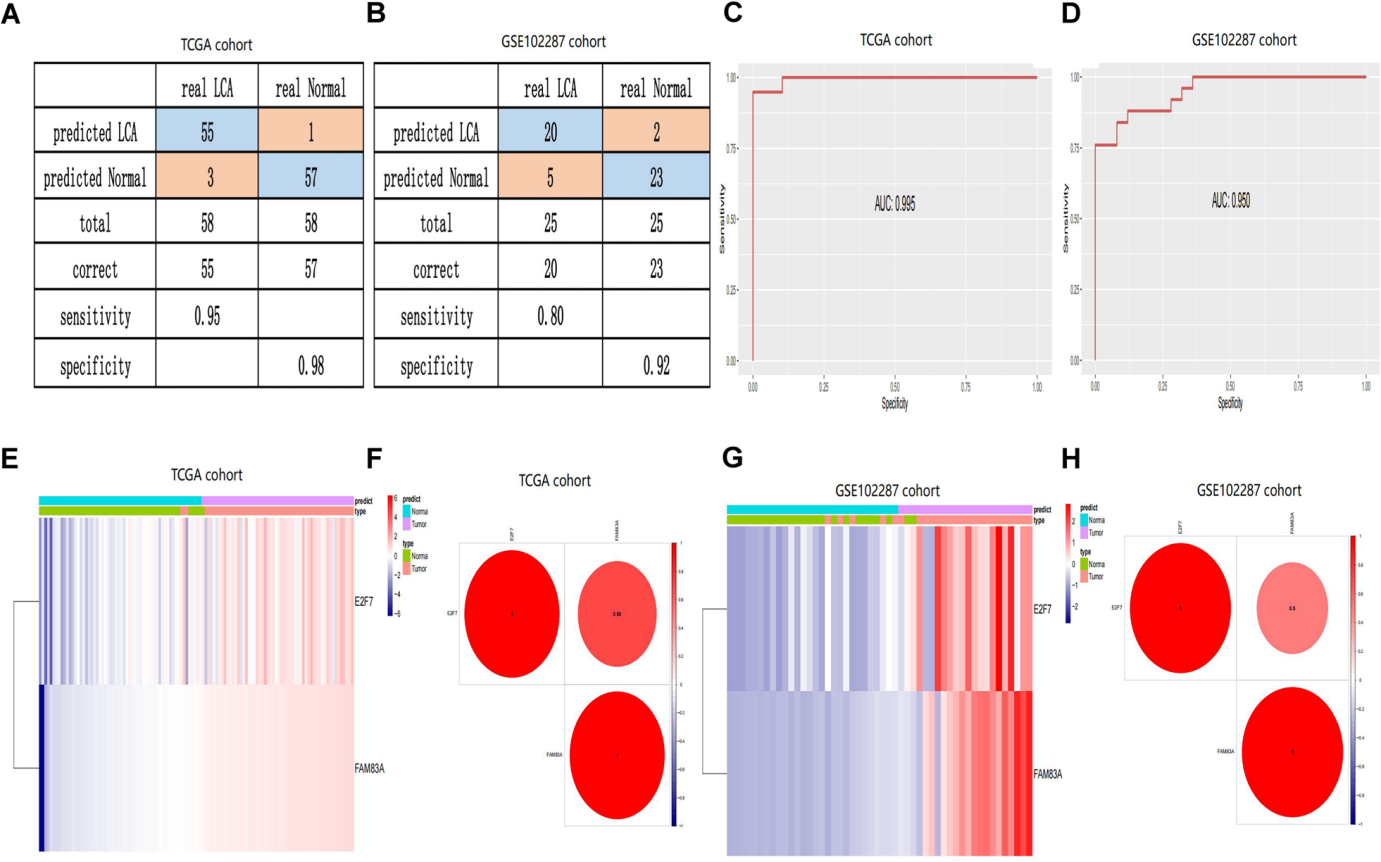
The diagnostic model effectively differentiates tumors from normal samples. **A**, **B** Confusion matrix highlighting the binary classification outcomes of the diagnostic model in both the training group (**A**) and the validation group (**B**). **C**, **D** ROC curves are employed to evaluate the predictive capability of the diagnostic model in the training (**C**) and validation (**D**) groups. **E**, **G** Unsupervised hierarchical clustering analysis of two cisplatin-related genes is conducted for the diagnostic model in the training group (**E**) and the validation group (**G**). **F**, **H** Analysis of the interrelationship among E2F7 and FAM83A is presented for the TCGA paired cohort (**F**) and the GSE102287 cohort (**H**)

The pressing requirement for a diagnostic model that aids in the early detection of LUAD for timely clinical intervention prompted the development of a model through stepwise logistic regression analysis. The training set included 58 normal and 58 matched LUAD samples from the TCGA database. For validation, 25 normal samples and 25 matched tumor samples were used from the GSE102287 dataset. As illustrated in Figs. 7A, B, the sensitivity and specificity were 0.95 and 0.98 in the TCGA cohort and 0.80 and 0.92 in the GSE102287 cohort. The AUC was 0.995 for the training set and 0.950 for the validation set (Fig. 7C, D). Additionally, Fig. 7E and G showed that the two genes could effectively distinguish LUAD samples from normal ones in unsupervised hierarchical clustering analysis. The correlation and combined effects of these two risk genes were further confirmed in both the TCGA and GSE102287 cohorts, as shown in Fig. 7F and H. In summary, our diagnostic model demonstrated high specificity and sensitivity in differentiating between LUAD and normal lung tissue.

### Knockdown of E2F7 inhibits cell proliferation and migration in LUAD cells

**Fig. 8:**
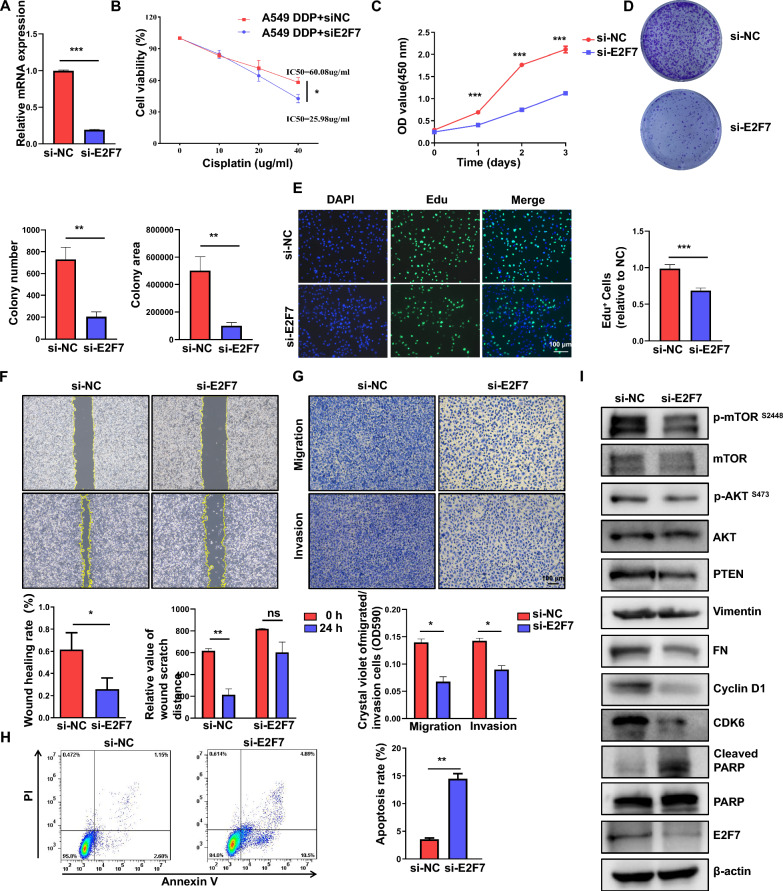
Knockdown of E2F7 inhibits cell proliferation and migration, while promotes apoptosis in A549 cells. **A** RT-PCR validation knockdown efficiency of E2F7 in A549 cells. **B** Cell viability was detected after E2F7 knockout in A549 cisplatin-resistant cells. **C**. CCK-8 assay showing the proliferative ability of A549 after E2F7 knockdown. **D** Colony formation assays of E2F7 knockdown in A549 cells. **E** Cell viability was determined by EdU assay. EdU^+^ cells are denoted in green, and nuclei are denoted in blue (Hoechst, 33,342). **F** Wound healing assay results showed that knockdown of E2F7 inhibited cell migration. **G** Migration and invasion of A549 cells transfected with control siRNA and E2F7 siRNA were evaluated by Transwell assays. **H** Flow cytometry results show that knockdown of E2F7 increased cell apoptosis. **I** Immunoblot detecting mTOR, AKT, PTEN, vimentin, FN, Cyclin D1, CDK6, PARP, and E2F7 expression in control and E2F7 silenced A549 cells. Data are shown as mean ± SD of triplicate measurements with similar results. *P < 0.05, **P < 0.01, ***P < 0.001

**Fig. 9:**
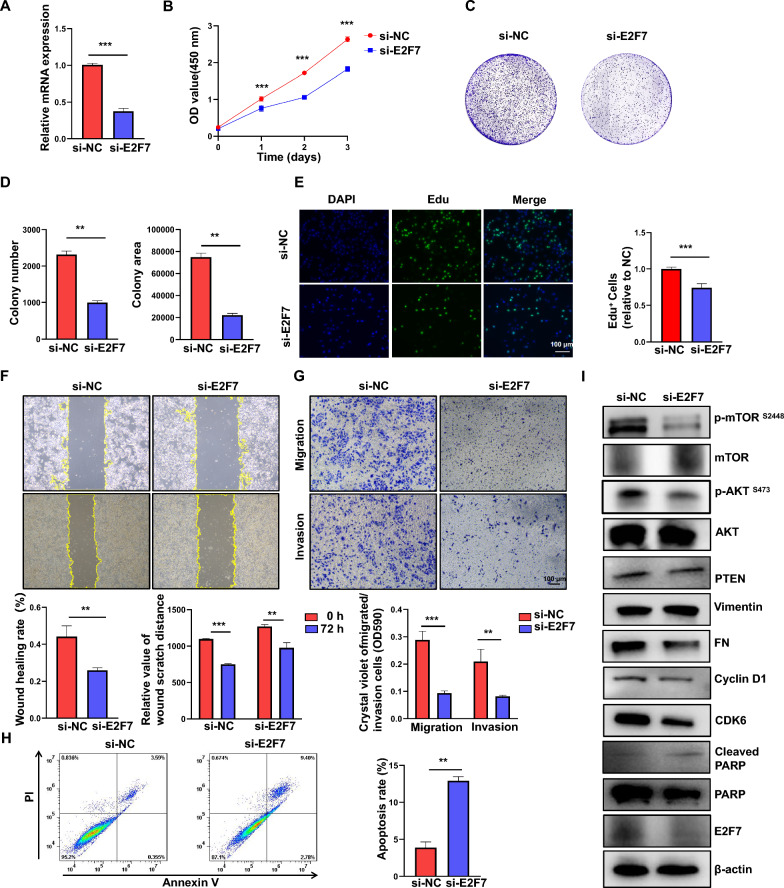
Knockdown of E2F7 inhibits cell proliferation and migration, while it promotes apoptosis in PC9 cells. **A** RT-PCR validation knockdown efficiency of E2F7 in PC9 cells. **B** CCK-8 assay showing the proliferative ability of PC9 after E2F7 knockdown. **C** Colony formation assays of E2F7 knockdown in PC9 cells. **D** Number of clones and measurement of the clone area in PC9 cells. **E** Cell viability was determined using the EdU assay. EdU^+^ cells are indicated in green, nuclei are indicated in blue (Hoechst, 33,342). **F** Wound healing results showed that knockdown of E2F7 inhibited cell migration. **G** Migration and invasion of PC9 cells transfected with control siRNA and E2F7 siRNA were evaluated using Transwell assays. **H** Flow cytometry results showing that knockdown of E2F7 increased cell apoptosis. **I** Immunoblot detecting mTOR, AKT, PTEN, vimentin, FN, Cyclin D1, CDK6, PARP, and E2F7 expression in control and E2F7 silenced PC9 cells. Data are shown as mean ± SD of triplicate measurements with similar results. **P < 0.01, ***P < 0.001

FAM83A plays a crucial role in regulating lung cancer proliferation, colony formation, and invasion [31–36]. Decreasing FAM83A levels using siRNA/shRNA cultured suppresses cell proliferation and induces cell apoptosis. Additionally, cell mobility is markedly reduced following FAM83A silencing, which leads to notable inhibition of subcutaneous tumor growth and lung metastasis in vivo [37]. FAM83A facilitates the proliferation and invasion of lung cancer cells by affecting the Wnt and Hippo signaling pathways and the epithelial-to-mesenchymal transition (EMT) process in A549 and H1975 cells [38]. E2F7 has been identified as a cancer-promoting gene in glioblastoma, liver cancer, and colon cancer [39–41]. However, its role in LUAD remains unclear. E2F7 was silenced in A549 cisplatin-resistant cells (A549/DDP) and PC9 cells (Fig. 8A, 9A), finding that E2F7 knockdown restores the sensitivity of A549/DDP cells (Fig. 8B). A CCK8 assay was used to evaluate cell proliferation, revealing that E2F7 knockdown significantly inhibits the proliferation (Fig. 8C, 9B) of A549 and PC9 cells. Knocking down E2F7 also suppresses colony formation (Fig. 8C, 9B, D). The EDU experiment confirmed that E2F7 knockout inhibits the proliferation of A549 and PC9 cells (Figs. 8E, 9E). Additionally, this study examined E2F7's role in regulating LUAD migration and invasion, finding that its knockdown suppresses A549 and PC9 cell migration and invasion (Fig. 8F, G, 9F, G). Furthermore, this study examined the ability of E2F7 to regulate cell apoptosis. The annexin V-PI staining results showed that E2F7 knockdown promotes apoptosis in A549 and PC9 cells (Fig. 8H, 9H). The cell proliferation, cell cycle, apoptosis, and EMT-associated proteins were assessed by western blot, observing decreased expression of p-mTOR, PTEN, Fibronectin, CyclinD1, and CDK6 after E2F7 knockdown, while cleaved PARP increased (Fig. 8I, 9I). These findings suggest that E2F7 knockdown effectively suppresses malignant behaviors in LUAD cells, highlighting its potential as a therapeutic target in lung cancer treatment.

### Knockdown of E2F7 suppresses lung cancer progression and metastasis in vivo

**Fig. 10:**
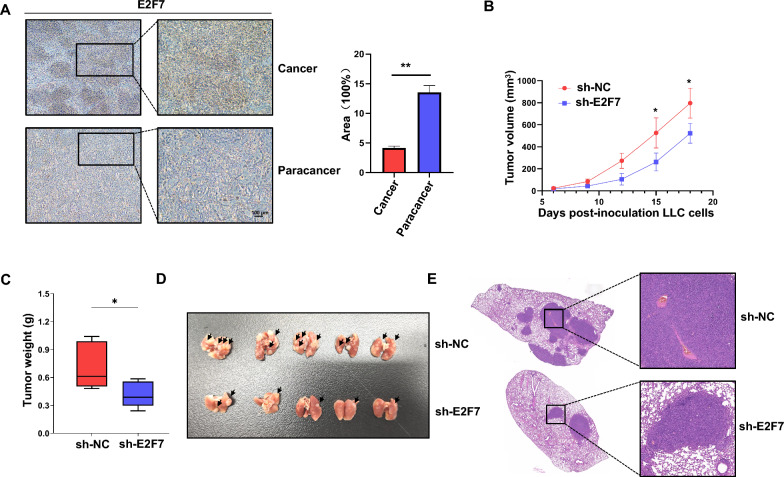
Knockdown of E2F7 suppresses LUAD progression and metastasis. **A** IHC was used to detect the expression of E2F7 in cancer and adjacent tissues in patients with LUAD. **B** Subcutaneous tumor growth curve statistics of the two groups (n = 5). **C** Subcutaneous tumor weight was computed in the two groups. **D** Images of the LLC cell lung metastasis model were treated with shNC and shE2F7 (n = 6). **E** H&E staining showing metastasis of LLC cells in the lung tissue. *P < 0.05

In another set of experiments, IHC staining detected high expression of E2F7 in the tumor areas of patients with LUAD, consistent with bioinformatics analysis results (Fig. 10A). The antitumor effect of E2F7 knockdown was evaluated in vivo using the LLC mouse lung cancer model. As shown in Fig. 10B, knockdown of E2F7 significantly inhibited tumor development, as indicated by the reduced weight of shE2F7 tumors compared to the control (Fig. 10C). To assess the effect on tumor metastasis, LLC cells were injected via the tail vein, resulting in fewer pulmonary metastatic foci in the shE2F7 group compared with the control group 20 days later (Fig. 10D). H&E staining confirmed that E2F7 knockdown markedly reduced lung metastasis of LLC cells (Fig. 10E). Together, these findings demonstrate that E2F7 knockdown significantly impedes lung cancer progression and metastasis, underscoring its potential as a therapeutic target in LUAD.

## Discussion

Lung cancer remains the leading cause of cancer-related deaths globally [1]. Platinum-based drugs, particularly cisplatin, play a critical role in treating lung cancer, but resistance to cisplatin significantly hampers chemotherapy success, often leading to more aggressive and metastatic disease [42]. While many genetic markers have been identified for their prognostic value in LUAD [43], the focus on cisplatin resistance-related genetic markers as predictors for immune response is lacking. Identifying new genes or gene combinations related to cisplatin resistance is vital for optimizing early diagnosis and treatment strategies, thereby enhancing treatment outcomes.

By examining transcriptome data of cisplatin-resistant LUAD cells from the GEO database, 181 genes associated with cisplatin resistance were identified. These genes are involved in pathways that facilitate tumor progression, such as "vascular development" and "transmembrane receptor protein tyrosine signaling." Using univariate regression analysis, random forest and multivariate regression analyses, two prognostic genes, E2F7 and FAM83A, were identified. These genes help segregate patients into low-risk and high-risk categories based on their median risk scores, correlating higher risk scores with increased mortality and shorter survival times. The dual-gene marker was also proven to be a reliable predictor of OS for LUAD patients and was confirmed as an independent prognostic factor through Cox regression analysis. Finally, we validated the prognostic accuracy of our gene markers with corresponding histological figures, establishing their strong predictive value for the prognosis of LUAD patients.

This study developed a prognostic model utilizing E2F7 and FAM83A as key indicators. The effect of FAM83A on LUAD tumor development has been widely studied, demonstrating its significant effect on crucial cellular functions such as proliferation, autophagy, and apoptosis. These processes are essential for the growth and progression of LUAD. Specifically, FAM83A has been shown to accelerate lung cancer progression through modulation of the Wnt and Hippo signaling pathways. It also triggers epithelial-to-mesenchymal transition (EMT) in NSCLC through the PI3K/AKT/Snail pathway [44]. Research studies have examined the gene expression of FAM83A in 362 patients with NSCLC, discovering that FAM83A is notably overexpressed in patients with lung cancer, which correlates with a poorer prognosis [45]. Furthermore, the role of the microRNA/E2F7 axis in the development of LUAD has been highlighted. Specifically, the Circ-AASDH/miR-140-3p/E2F7 regulatory axis plays a crucial role in advancing LUAD, while the progression of NSCLC is effectively hindered by celastrol through its action on the circ_SATB2/miR-33a-5p/E2F7 signaling pathway. Additionally, E2F7's close association with the metastasis of small cell lung cancer has been documented. A recent study introduced a novel m7G score to measure the level of m7G modification in LUAD, basing the score on four genes (E2F7, FAM83A, HOXA13, and PITX3) to improve the assessment of chemotherapy and immunotherapy strategies. This research proposed a model for predicting the prognosis of LUAD and the response to immunotherapy through the lens of m7G,although the specific role of m7G in cancer development and its clinical relevance are yet to be fully elucidated [49]. To address this, our study conducted in vitro cell experiments to investigate E2F7's function in regulating LUAD tumor cells, revealing that silencing E2F7 significantly reduced cell proliferation, migration, invasion, and subcutaneous tumor growth, thereby confirming E2F7's involvement in LUAD and the precision of our analysis methods.

To investigate the biological roles of E2F7 and FAM83A further, this study performed GSEA, which indicated their involvement in metabolic pathways, including those for cysteine, methionine, glucose, fructose, mannose, purine, and pyrimidine. The interaction between these genes and metabolic molecules, and the potential regulatory effects of these molecules on the genes, warrant further study with clinical samples in metabolomics research.

Moreover, the significance of tumor-infiltrating immune cells in cancer dynamics and response to treatment is increasingly recognized. The advent of immune checkpoint inhibitors has transformed the therapeutic landscape for metastatic lung cancer, with notable successes. Yet, the effect of cisplatin resistance on the immune microenvironment is still to be understood. Our research examines the association between gene features, differential gene expression, and the immune microenvironment. The results showed that, compared with low-risk patients, those at high risk showed greater infiltration of CD4 memory-activated T cells and CD8^+^ T cells, suggesting a potential higher benefit from immunotherapy for this group. Our findings indicate a pro-inflammatory microenvironment in high-risk patients with LUAD, prompting further investigation into the underlying mechanisms.

Cancer cells avoid immune system detection by utilizing inhibitory pathways, notably through the increased expression of checkpoint genes. Our analysis examined the relationship between specific gene features and immune checkpoint genes. The findings indicated that the expression of PD-L1, PD-L2, B7H3, and IDO1 was higher in patients within the high-risk category. This suggests these patients might have stronger pro-tumor immune responses, potentially contributing to their poorer prognosis. Research into tumor immune escape mechanisms has shown that ICIs are effective in treating lung cancer, with the treatment response closely linked to the expression of immune checkpoints within the tumor environment. Thus, linking our prognostic model to immune checkpoint expression levels could aid in identifying patients likely to benefit from ICIs.

This study performed in vitro experiments to demonstrate that reducing E2F7 levels has anti-cancer effects on LUAD, achieved by hindering cell growth through the AKT/mTOR signaling pathway and increasing apoptosis signals. Moreover, in vivo tests indicated that lowering E2F7 levels could reduce the growth and spread of lung cancer tumors. Overall, our research offers new potential treatment and management strategies for patients with LUAD. However, the in vivo studies used mouse cells, necessitating further research with human lung cancer cell xenografts in mice.

Our LUAD research provides valuable insights but also acknowledges limitations: (1) Data source limitations, with primary data from the TCGA and GEO databases requiring additional cohorts for more robust external validation, especially for immunotherapy. (2) Insufficient Clinical Samples, with a limited number of LUAD clinical samples affecting our ability to evaluate different prognostic models due to missing tumor staging data. (3) The role of E2F7 in the immune microenvironment was not thoroughly explored, focusing mainly on tumor cell responses and cisplatin resistance. Future work will delve into E2F7's role in the cisplatin-resistant LUAD tumor environment. (4) Animal experiments and regulatory mechanisms need further exploration to understand E2F7's regulatory mechanisms and confirm our prognostic model's accuracy through prospective data analysis.

### Supplementary Information

Supplementary Material 1. Table 1 The DEGs information of cisplatin-resistance and non-resistance LUAD cohorts.

Supplementary Material 2. Table 2. Univariate regression analysis of cisplatin-resistance and non-resistance LUAD cohorts.

Supplementary Material 3. Table 3 Random forest of cisplatin-resistance and non-resistance LUAD cohorts.

Supplementary Material 4. Table 4. Primers used for qRT-PCR. Supplementary Table 5: Antibodies used for western blotting.

## Data Availability

The other datasets supporting the conclusions of this article are included within the article and its additional files. Further information and requests for resources and reagents should be directed to and will be fulfilled by the corresponding author, Yun Xu (11618103@zju.edu.cn).

## Abbreviations

AUC: Area under the curve
C-index: Concordance index
CI: Confidence interval
CTLA4: Cytotoxic T lymphocyte-associated antigen-4
DEGs: Differently expressed genes
EMT: Epithelial-to-mesenchymal transition
EdU: 5-Ethynyl-2’-deoxyuridine
E2F7: E2F transcription factor 7
FAM83A: Family with sequence similarity 83 member A
GSEA: Gene set enrichment analysis
GPX2: Glutathione peroxidase-2
HR: Hazard ratio
ICI: Immune checkpoint inhibitor
LUAD: Lung adenocarcinoma
MEKi: MEK inhibitors
MAPK: Mitogen-activated protein kinase
NSCLC: Non–small cell lung cancer
OS: Overall survival
PFS: Progression-free survival
PBS: Phosphate-buffered saline
PD-L1: Programmed death-ligand 1
PD-1: Programmed death-1
PCA: Principal component analysis
ROC: Receptor operating characteristic
TLR9: Toll-like receptor 9
