# Supplementary material for: Integrated analysis reveals critical cisplatin-resistance regulators E2F7 contributed to tumor progression and metastasis in lung adenocarcinoma
Source: Cancer Cell Int. 2024 May 17;24:173. doi: 10.1186/s12935-024-03366-6 (PMC11102206; doi:10.1186/s12935-024-03366-6)
Supplement: Supplementary file 4 — Supplementary Material 4. Table 4. Primers used for qRT-PCR. Supplementary Table 5: Antibodies used for western blotting. [file 12935_2024_3366_MOESM4_ESM.docx]

**Supplementary Material**

**Integrated analysis reveals critical cisplatin-resistance regulators E2F7 contributed to tumor progression and metastasis in lung adenocarcinoma**

**Supplementary Table S4.** **Primers used for qRT-PCR.**

| **Target** | **Forward** | **Reverse** |
| --- | --- | --- |
| E2F7 | CTGCTGCGCTAGACTTGGAT | TCTCTTAGTAGGACCACCAACG |
| 18S | GAGGTAGTGACGAAAAATAACAAT | TTGCCCTCCAATGGATCCT |

**Supplementary** **Table S5: Antibodies used for Western blotting.**

| **Primary Antibody** | **Source** | **Catalog #** | **Dilution** |
| --- | --- | --- | --- |
| CyclinD1 | **CST** | 55506 | **1:1000** |
| CDK6 | **CST** | 13331 | **1:1000** |
| AKT | **CST** | 4685 | **1:1000** |
| phospho-AKT | **CST** | 4060 | **1:1000** |
| mTOR | **CST** | 2983 | **1:1000** |
| phospho-mTOR | **CST** | 5536 | **1:1000** |
| PTEN | **CST** | 9188 | **1:1000** |
| Vimentin | **CST** | 5741 | **1:1000** |
| cleaved PARP | **CST** | 5625 | **1:1000** |
| PARP | **CST** | 9532 | **1:1000** |
| FN | Abcam | ab2413 | **1:1000** |
| E2F7 | Proteintech | 24489 | **1:1000** |
